# Swine influenza A (H1N1) strikes a potential for global disaster

**DOI:** 10.4103/0974-2700.50744

**Published:** 2009

**Authors:** Sagar Galwankar, Angela Clem

**Affiliations:** Department of Global Health, University of South Florida, 13201 Bruce B. Downs Blvd., MDC 56, Tampa, FL 33612, USA

**Keywords:** Pandemic, pigs, populations, prevention, public health

## Abstract

As of April 25^th^2009, 11.00 AM, eight human cases of swine influenza A virus infection have been identified in the United States in California and Texas. There is also established evidence of similar cases across the United States border in Mexico. Experts from the Centers for Disease Control and Prevention in cooperation with World Health Organization and public health experts from Canada and Mexico are leading an exhaustive investigation to find the source of infection and infected people. We present a profile of this illness from the available literature.

## INTRODUCTION

We have now confirmed eight cases of genetically similar swine influenza virus (SIV) A (H1N1) viruses in human patients from the United States. As per current investigations, these viruses have a genetic structure which is not similar to the known genetic make up of swine or human influenza viruses.[1] Swine flu is known to be transmitted by pigs to humans; however, this does not appear to be the situation with these diagnosed cases; and, it is suspected that interhuman transmission might have occurred. The influenza vaccine against the H1N1 strain is unlikely to be effective in establishing immunity against this infection.

## HISTORICAL PERSPECTIVES OF INFLUENZA

The last 400 years have seen regular influenza virus outbreaks of people suffering from respiratory illness.[2,3] The year, 1580, marks the first instance of influenza recorded as an epidemic; even though, there is a possibility that there were many prior influenza epidemics. From 1918–1919, there were waves of influenza outbreaks which resulted in nearly 21 million deaths across the world of which approximately half a million were in the United States.[4]

In 1933, Smith and his team isolated influenza A virus from ferrets.[5] Francis isolated influenza B virus 1939; and, finally, Taylor isolated influenza C in 1950.[6,7] From 1936 to 1950, extensive studies led to the discovery that influenza virus could be grown in embryonated hen eggs. This allowed for the development of vaccines and to the discovery of the phenomenon of hemagglutination, which then led to the creation of the easy and cost effective methods for measurement of viral antigen and antibody.[8–10]

In 1977, influenza A (H1N1) viruses produced epidemic disease in children and young adults worldwide. This viral strain was similar to the outbreaks prior to 1957. It is theorized that people born before 1957 likely had developed immunity to the virus after getting exposed to it. Thus, the majority of the cases were from the younger generation who had not previously been exposed to the A/H1N1 infection. By 1978, the virus had spread worldwide and had not spared the United States. For this reason, this strain of virus was included in the 1978–79 vaccine.[11]

A live vaccine against influenza was licensed in the United States in 2003. Finally, four antiviral medications were approved for preventing and treating influenza. These antivirals include the M2 inhibitors, amantadine (1960's), and rimantadine (1993), and the newer neuraminidase inhibitors, zanamivir, and oseltamivir (2000). Although the neuraminidase inhibitors are clinically active against both influenza A and B, the M2 inhibitors are active only against influenza A.[12]

## BASIC OVERVIEW OF INFLUENZA[13]

The three types of influenza viruses are A, B, and C. They belong to the Orthomyxoviridae family of single stranded RNA viruses. Type A viruses are subtyped on the basis of two surface glycoproteins, hemagglutinin (HA), and neuraminidase (NA). Furthermore, the influenza A subtypes and the influenza B viruses are further classified into strains. Type A influenza viruses are further classified based on differences in the hemagglutinin (HA) and neuraminidase (NA) proteins found on the surfaces of the influenza viruses. There are 16 known HA subtypes and 9 known NA subtypes of influenza A viruses, which can recombine to create novel combinations of influenza.

Two phenomenon of great importance that can lead to the occurrence of novel strains of influenza virus are antigenic drift and antigenic shift. When the seasonal influenza viruses undergo point mutations which modify their genomes, it is called antigenic shift. The typical seasonal influenza viruses exhibit frequent point mutations that lead to more gradual shifts in their genomes. This process is known as antigenic drift; and, it is the reason that new influenza vaccines must be prepared each year. On the other hand, if there is a reassortment of the gene segments leading to the development of novel influenza A viruses, it is known as antigenic shift.

Influenza B and C viruses are only found in humans, and do not possess a pandemic potential. Influenza viruses can be highly resilient in the environment. These viruses can survive in contaminated manure for at least three months in cool climates. The virus can survive in water for more than 72 h at 72°F, greater than one month at 32°F, and potentially indefinitely in frozen materials. Influenza A viruses can infect a variety of animals including pigs, whales, horses, seals, and humans however; there have been past instances of crossing the species barrier.

## HISTORICAL PERSPECTIVES OFSIV IN PIGS

In 1930, the classical swine flu virus Type A (H1N1) was first isolated from pigs.[14] In the last decade, pigs in North America have seen the emergence of three different subtypes and five different genotypes of novel influenza viral infections amongst its swine communities. This phenomenon was noticeable because the past six decades had seen no major changes in the overall epidemiological trends for SIV in North America. Classical H1N1 viruses were only seen in pigs up until 1991 when antigenic drift variants of these H1N1 viruses emerged. The real antigenic shift occurred with the emergence of H3N2 viruses around 1991. There were H3N2 outbreaks among pigs in the United States. This virus was previously found in humans. The genes of these H3N2 viruses were derived from human, swine, and avian viruses and since then have become a major cause of swine influenza in North America. Furthermore, the reassortment between the H3N2 viruses and classical H1N1 swine viruses has resulted in H1N2 viruses which have infected pigs. More recently in 1999, the avian H4N6 viruses crossed the species barrier to infect Canadian pigs. These continuous antigenic shifts could have led to a sudden antigenic drift which crossed over to humans to create the potential for the current global disaster.[15]

## SIV INFECTION PROFILE IN PIGS

Pigs get SIV induced respiratory disease from a Type A influenza virus. This has caused regular outbreak of swine infections which have a very low death rate. Like in humans, the SIV infections occur in winter months. The swine virus mutates regularly causing antigenic shifts along with the mixing of the avian and human variants to which the pigs are also susceptible. Over years many variants of the Type A Influenza viruses have been isolated the latest being the H1N1 viruses.

Close contact amongst pigs leads to transmission. Herd immunity may play a role of sporadic diseases outbreaks in pig communities which have regular outbreaks. Fever, change of behavior, coughing, nasal and eye discharge, shortness of breath and anorexia and refusal to eat may be signs of infection.

Currently pigs are vaccinated to prevent swine influenza but there is no vaccine against human infection. The human influenza vaccine does not protect against the swine H1N1 viruses.[15]

### Crossing species

Pigs are susceptible to human and avian influenza A viruses; hence, they are believed be effective intermediate hosts for the evolution of new viruses with a potential to produce pandemics. This occurs because the swine serves as a melting pot to generated new hybrid viruses after genetic resortment. Recently a case of SIV in a human was reported in Europe. This case documented the probability of the human infection secondary to this novel and new swine influenza virus.[16] A group of researchers reported that in 2008, two swine H5N2 viruses were isolated from Korean pigs whose genetic analysis revealed that they originated from avian influenza viruses. This virus was an assortment of the swine and avian virus with some of its genes namely PB2, PA, NP, and M genes derived from the Korean swine H3N1 virus. This virus was found to be more virulent and easily transmissible in pigs. It was anticipated that such changes in the genome of the virus could lead to human transmission which could have grave results because of the lack of immunity and absence of vaccine.[17]

## CURRENT EPIDEMIOLOGICAL INVESTIGATION

As per the CDC their Epidemiologists and Experts are trying to follow leads in California, Texas, and Mexico. There are established cases of influenza A in the United States and isolates from patient specimens in Mexico have also tested positive for the identical strain of swine influenza A (H1N1) at the CDC laboratory. Six of the eight cases in the United States are only sensitive to zanamivir and oseltamivir. There are a large number of patients in Mexico who are infected; and, all the agencies are working with the Mexican government to institute the maximum number of strategies to contain this virus.[1]

## SIV IN HUMANS

Historically, human have been sporadically infected with swine flu. Primarily, these patients were directly exposed to pigs while working in swine industry or living on farms with pigs. Since 2009, more than ten cases of swine infection in human infection have been reported.

The symptoms of swine infection range from fever and anorexia to coughing, rhinorhea, and gastrointestinal upset. The ingestion of pork and pork products does not transmit the virus. Pneumonia, respiratory failure, and deaths have been reported with swine flu infection in humans; and, as with seasonal influenza outbreaks, SIV in humans can worsen underlying chronic conditions. The use of proper handling techniques and cooking pork to a temperature of 160°F makes it safe to be consumed.

Transmission of the influenza viruses can occur between humans and pigs. This transmission occurs primarily when the pigs live in proximity to each other like pigs on a farm or in the pig industry. Human to human transmission can occur as with any other influenza infection.[15]

The main route of transmission in humans is via inhalation of infected respiratory droplets after coughing and sneezing. Influenza viruses typically range in size from 0.08 to 0.12 µm. Fomites like infected surfaces and materials can also transmit the virus between humans.[13]

## DIAGNOSIS

The CDC defines a confirmed case of SIV in humans as a person who has acute respiratory symptoms and who has tested positive for swine influenza A (H1N1) virus infection at a CDC laboratory with one of the following tests namely the real-time RT-PCR, viral culture or an established four-fold rise in SIV A (H1N1) specific neutralizing antibodies.[18]

The specimen needs to be collected within the first 200 h of illness when it most likely that the patient is shedding the virus. It also known that children may shed the virus up to 10 days or more. In the United States, the CDC laboratories expect the sample to be sent to them for testing.

## PREVENTIVE SOCIAL AND BEHAVIORAL STRATEGIES[19]

Strategic recommendations for the prevention of SIV are being developed at all levels. Healthy people, people in health care, and the infected individuals are all a part of this process to contain and prevent the spread of this threat. The prevention of human to human transmission is the key to preventing a swine influenza pandemic.

Healthy people should continue to practice behaviors which ensure that they remain healthy. Covering your mouth and nose with a tissue while sneezing and then discarding the tissue in a trash container is recommended. This should be followed by washing your hands with soap and water or even cleaning them with alcohol to ensure effective infection prevention. Individuals should avoid touching their nose, mouth, and eyes because these droplets can spread easily and cause infection.

It is recommended that contact with sick people be avoided because the infection spreads by droplets from sneezing or coughing. If you have a respiratory infection then it is advised that you stay at home and restrict your social contacts.

The CDC has recommended that residents of California or Texas with symptoms of fever, upper respiratory infection, sore throat, nausea, vomiting, and/or diarrhea should contact their physicians to determine a further course of action.

As stated above, people who are sick with an upper respiratory infection or any of the above listed symptoms should try to stay away from healthy people because the virus can be transmitted while the symptoms last and possibly even seven days following the onset of illness. This infectious period can longer in younger children.

## SEEKING EMERGENCY CARE[19]

Patients should seek immediate care if they have difficulty in breathing, develop a bluish skin color or rash-like redness, generalized weakness, severe anorexia, chest pain, abdominal pain, worsening headache, and/or confusion. In children, decreasing oral intake, worsening fever, increasing irritability, increasing lethargy, non-remitting fever, a bluish skin color or rash-like redness, and/or difficulty in breathing are all signs which require emergency care. Severe nausea, vomiting, or diarrhea which can lead to dehydration can also be indicative of SIV in humans.

## RECOMMENDATIONS FOR CLINICIANS[20]

Human swine influenza should be considered a differential diagnosis for patients with febrile upper respiratory tract infection with or without gastrointestinal symptoms. For clinicians practicing in the United States the suspicion for SIV human infection should arise when they see patients who live in the affected areas of California or Texas or who have travelled there and patients who travelled recently to Mexico or who were been in contact with persons who had febrile respiratory disease either in Mexico or the United States in the past week. The physician should obtain a swab from the nasopharynx from suspected patients and place the swab in a transport medium made only for viruses. The physician should then refrigerate the specimen and immediately contact the local or state health department to facilitate the transport of the specimen to a designated state or federal laboratory like the one at CDC. State laboratories have been instructed to immediately transport all specimens of untypable influenza A to the Influenza Division's viral laboratory at the CDC.

## RECOMMENDATIONS FOR HOME-BASED CARE PROVIDERS[21]

Care providers of patients who are suspected to have SIV or who are diagnosed with SIV but are being cared at home should be prompt in isolating themselves and the patient from social contact until the patient is free from the symptoms or until the physician clears them. The patient should be isolated in a separate room with the door always closed. The patients should not be allowed to leave the house; but, if they have to leave then they should be instructed to practice preventive behavioral strategies and to cover their nose and face with a surgical mask. This recommendation applies even when the person is leaving his/her room, using the common areas of the house, or going to the bathroom. It recommended that there be a separate bathroom for the patient, but if this is not possible then the bathroom should be cleaned daily with commonly available disinfectants and cleaners. All laundry in the house should be individualized as the infection can be transmitted by fomites. Adequate ventilation should be maintained in the house in the form of keeping windows open for prolonged periods of time.

The physician should be closely involved if there is a need for directions to manage pregnant patients and infected children of patients with chronic debilitating conditions. If the care providers get infected, they should follow similar precautions. Please seek emergency care if any of the signs as mentioned above appear in your patients or you notice them in yourself. The rules of isolation, hand washing and practicing clean behavior apply to you, patient, and all the family members in the house. Children should not go to school when ill. Pregnant patients should stay away from patients or people suspected to have the illness. The caregiver should also wear a mask when caring for an infected or suspected infectious loved one. This is important because the caregiver can be infected and can spread the infection to others.

## ENVIRONMENTAL PREVENTION STRATEGIES[22]

In addition to isolation and behavioral modifications, wearing a face mask is equally important in prevention of the disease. Two types of masks namely the simple surgical mask and N95 respirator are recommended for use. The choice between using a simple surgical mask and a N95 Respirator is a personal one. A N95 respirator can filter out small particles which are not filtered by a face mask. After use, these facemasks should be properly disposed of in a sealed trashcan. It is recommended that when caring for an infected person who is using a nebulizer the N95 mask is better suited for protection against exposure. Reuse of these face masks is not recommended due to the potential for exposure. There are some facemasks which are reusable after they are washed and thoroughly dried; however, please practice all the recommended behavioral interventions after using these masks.

Droplet precautions and preventing fomite-based transmission is an important part of environmental strategies. All paper tissues used by the patient should be disposed in the trashcan. All furniture should be cleaned with disinfectants. Linens, utensils, and bathrooms need to be cleaned daily. It is recommended that utensils and other items of daily use not be shared with an infected individual. All utensils should be thoroughly cleaned with soap and warm water .Frequent laundering of linens is highly recommended in a house where there is an infected person. All these linens should be washed well and dried.

## BIOSAFETY OF LABORATORY PERSONNEL[23,24]

Biosafety Level 2 (BSL-2) laboratories are used to diagnose and isolate the influenza virus A. In contrast, the BSL-1 laboratory is only suitable for working with microbes not known to cause human infection. A typical BSL-1 lab is found in high schools and colleges teaching microbiology classes and the biological agents involved are not considered a hazard. A BSL-2 lab mandates policies and practices geared to create safety levels for personnel dealing with biological agents with moderate risk to humans. Normally these are agents seen in the community; and, the workers may already be immune to them. For more hazardous organisms, BSL-3 labs deal with organisms which can be aerosolized and cause lethal disease in humans. Personal protective equipment, N95 Masks, shoe covers, tight gown, double gloving on each hand and eye protection are mandatory in the laboratory. Disposal of biological waste should be done according to the procedures set by the laboratory. 70% ethanol, 5% lysol and 10% bleach are all appropriate as disinfectants. Further, all laboratory staff should be educated regarding the signs of disease; and, antiviral prophylaxis should be considered for the seven days after exposure.

## INFECTION CONTROL IN HOSPITALS[25,26]

A confirmed case of swine influenza A (H1N1) virus is defined as a human patient diagnosed at a CDC laboratory by any one of the following tests including real-time RT-PCR, viral culture or a four-fold rise in swine influenza A (H1N1) specific neutralizing antibodies. A suspected case of swine influenza A (H1N1) virus infection is a patient who in the previous seven days has come in contact with a confirmed case. A close contact person is one who has been within six feet of a confirmed or suspected case.

The most frequent presenting symptoms of swine influenza will be indicative of an acute respiratory illness. More specifically, symptoms of this infection may include rhinorrhea, sore throat, cough, fever or nasal congestion. The presentation of at least two of the above symptoms can indicate the existence of an acute respiratory illness.

Patients are considered contagious up to the first week following onset of illness. If the illness continues beyond the first week, these patients should be considered contagious and should be isolated until symptoms resolve. Children may be contagious for a longer duration. Further, there is not a well-documented incubation period for the swine influenza A (H1N1) virus strain in humans. If patients are staying at home, they should exercise all the behavioral, social and environmental strategies and also enforce them for their family members and care providers. It is always recommended to seek emergency care if there is any clinical worsening of the symptoms.

Strict isolation precautions in an airborne isolation room should be enforced for confirmed cases of SIV A (H1N1) in humans. A High Efficiency Particulate Air (HEPA) filter air exchange system with at least 12 air changes per hour in a negative pressure room is necessary to provide adequate isolation and containment. All behavioral, biosafety, isolation, and environmental strategies are required to contain the illness and isolate the patient. The patient cannot leave the room; and, the bathroom must be attached to the room. Wearing protective gear, cleaning laundry, room and utensils and isolating all the contact fomites is essential to ensure standard, droplet and contact precautions. Conjunctival exposure should be prevented by wearing goggles in addition to gowns, gloves, head covering, and N95 masks. Biodisposal should be done as per practices mandated by the hospital. And, respirator use should be in accordance with the Occupational Safety and Health Administration (OSHA) regulations.

## PANDEMIC POTENTIAL AND PREPARATION FOR SWINE INFLUENZA[27]

Pandemics start when new virus subtypes emerge. These new viruses can infect humans and easily transmit between individuals causing serious illness and death. Because pandemics are global outbreaks, it is essential that there be adequate preparation to contain and prevent further damage. Some of the recommended initiatives are listed in Table 1.

**Table 1:** Directives for pandemic preparation

Develop preparedness plans to anticipate widespread illness and insufficient supplies
Participate and promote public health efforts
Communicate with healthcare providers regarding signs and symptoms of an outbreak
Implement prevention and control actions recommended by healthcare providers
Encourage sick employees/students to stay home to decrease the transmission of swine
Develop plans for the possibility of having a significant portion of workers/students absent due to illness
Practice good health and hygiene habits
Stay informed about pandemic influenza and be prepared to respond

### Surveillance for swine influenza[13]

It is highly important that epidemiological surveillance is carried out to detect the presence of swine influenza in both swine and humans so that potential outbreaks can be prevented and the spread of disease can be contained. Sentinel surveillance and syndromic surveillance are both very effective strategies for rapidly detecting swine influenza in a community.

In the sentinel provider system, information is gathered from recruited healthcare providers who report the number of influenza-like illnesses that they have treated weekly. These figures are then used to draw percentages for patient visits with influenza or influenza-like illness. In addition, because swine influenza is originating from pigs it may possible to adapt the poultry surveillance system in which poultry are regularly tested for the virus. This method could provide an early warning that swine influenza has infected pigs and that it has reached a community. This will alert the surveillance system to look for infected patients. Such an initiative has been very effective for arbovirus surveillance using chickens.

The syndromic surveillance uses a diagnostic code based system where the codes are put into a patient's medical records by the physician during the treatment process. Each of these codes is linked to 13 syndromes. For identifying an influenza-like illness, at least one of a set of respiratory illness codes and a fever of at least 100°F must be indicated in the medical record. This information is collected from the medical records daily and quantified by area codes, pin numbers, or ZIP codes. The results are then analyzed statistically; and, the cluster data can detect an influenza-like illness in a particular area.

### Vaccination strategy

There are vaccines which can prevent swine influenza in pigs; but, there is no vaccine to protect humans from swine flu. The seasonal influenza vaccine may provide partial protection against swine flu H3N2 viruses, but not the swine H1N1 viruses.[14]

As per the St. Petersburg Times of 26^th^ April 2009 “A “seed stock” genetically matched to the new swine flu virus has been created by the Centers for Disease Control and Prevention. If the U.S. government decides that vaccine production is necessary, manufacturers would need that stock to get started. The CDC warned that it would take months before enough doses for all Americans are ready.” This leaves prevention methods to play a major role in containing the virus.

## TREATMENT RECOMMENDATIONS[27]

The CDC has created a fact sheet for treating various groups of the population and has developed recommended guidelines for providing prophylaxis to suspected or exposed cases. In children, aspirin or aspirin related drugs should not be used in a confirmed or a diagnosed case of swine influenza A (H1N1) virus infection due to the risk of Reye syndrome. Instead, acetaminophen or non-steroidal anti-inflammatory drugs can be used to control the fever associated with the viral syndrome.

Like the human influenza A (H3N2) viruses, the swine influenza A (H1N1) virus is susceptible to both neuraminidase inhibitors, zanamivir, and oseltamivir. The human influenza A (H1N1) viruses are sensitive to zanamivir, amantadine, and rimantadine. Current recommendations call for considering co-infections with human and swine viruses, which have different drug susceptibilities. It is therefore recommended that a suspected case should be treated with zanamivir in combination with any of the three drugs. A confirmed case can be treated with zanaminir or oseltamivir. The treatment should be given for 5 days. When it comes to treating pregnant patients, all the above drugs are listed as “Pregnancy category C” because they have not undergone clinical studies to assess their safe use during pregnancy. It is therefore necessary that these drugs be used with caution. They should only be used if their potential benefits justify the risks to the fetus. Even though amantadine and rimantadine have demonstrated teratogenicity in animal studies, no adverse effects were reported from the pregnant women or their fetuses who were administered these drugs.

### Antiviral chemoprophylaxis

Prophylaxis should be considered for all suspected cases that have been exposed to a confirmed case. These exposed cases could include children in schools of high prevalence areas, travelers to Mexico, household and family contacts of patients, healthcare workers, first responders, emergency personnel, elderly individuals, and all individuals with chronic medical diseases. The antivirals, oseltamivir, and zanamivir, are recommended for chemoprophylaxis against swine influenza A (H1N1) virus infection. This prophylaxis can be given for a week after exposure to a confirmed case.[27]

## TRAVEL ADVICE WHEN TRAVELLING TO HIGH PREVALENCE AREA[29]

The CDC has not recommended restricting travel to Mexico but requests that travelers be aware of media updates and continuously monitor the global situation of the disease. The most updated information is available on the CDC website. Travelers are encouraged to visit a travel medicine physician to seek specific directions regarding their travel to a high prevalence area. High-risk patients suffering from chronic diseases like cancer, asthma, diabetes, advanced age or being accompanied by children are recommended to discuss with their doctor about carrying oseltamivir or zanamivir with them on the trip as the seasonal influenza vaccine is not protective towards the current swine virus infecting humans. These drugs help in decreasing the intensity of the illness in the event that a patient gets infected. They also assist in preventing complications and in hastening recovery. These medications work very well if started within the first 48 h. It is advised that emergency care be sought if there is any worsening of symptoms. Travelers are requested to update all of their vaccinations, which include the seasonal influenza vaccine. Travelers should always carry a medical supply kit and gain knowledge about the health resources available at your travel destination. It is further recommended that travelers should purchase International Travel Insurance which covers evacuation in the event of sickness and for the travelers to have the addresses of their consulates and embassies with them. Travelers should also be aware of the public health rules regarding the diseases in the area to which they are travelling because the rules may be different from the ones practiced in their geographic region. Remember to practice all the prevention strategies and to seek medical care when needed. On returning back home, travelers who believe they are infected or have been potentially exposed to an infected patient should contact their physician immediately. It is prudent that travelers describe all the details of their travel history to their doctor who can then decide on the future course of action.

The St. Petersburg Times on 26^th^ April 2009 reported that “WHO officials were discussing whether to declare an International Public Health Emergency, a move that could involve travel advisories and closing the borders. It has three criteria necessary for a Global epidemic to occur: The virus is able to infect people. It is readily spread person to person. And, the global population has no immunity to it”.

## DISPOSING OF THE INFECTED CARCASSES OF HUMANS

As per the CDC Telephone helpline at 1700 h on 26^th^ April 2009, there was no information about the potential of swine influenza virus spreading from dead bodies; but, it was recommended that the dead bodies be properly covered in bags and disposed with proper biohazard precautions with adequate protection to the disposers.[30]

These protocols would be similar to the ones employed during an infectious disease outbreak. Proper disinfection and personal protection devices should be used when disinfecting and disposing bodies, cleaning equipment and vehicles. Droplet, contact, and isolation precautions should be practiced in these situations. All prevention strategies used for preventing infection from the living can also be used for the dead until further information is available. There is no advisory on providing prophylaxis to health personnel exposed to the infected dead bodies; but, given the intensity of the situation, it would not be harmful if they were given prophylaxis. Cultural sensitivity should be an integral part of this disposition process.

## CONCLUSION

Swine influenza A (H1N1) has infected people in Mexico and the United States. Immediate strategies for containment have become the key factor in preventing the spread of this virus. With no vaccine in place, this novel influenza virus of swine origin needs drastic and intense precautionary measures to make it containable and preventable. The role of academies, researchers, educators, media, industry, and international agencies is crucial to ensure that people get the right information at the right time so that the outcome can be positive in this time of global fear and infectious crisis.
